# A checklist is associated with increased quality of reporting preclinical biomedical research: A systematic review

**DOI:** 10.1371/journal.pone.0183591

**Published:** 2017-09-13

**Authors:** SeungHye Han, Tolani F. Olonisakin, John P. Pribis, Jill Zupetic, Joo Heung Yoon, Kyle M. Holleran, Kwonho Jeong, Nader Shaikh, Doris M. Rubio, Janet S. Lee

**Affiliations:** 1 Division of Pulmonary, Allergy, and Critical Care Medicine, Department of Medicine, University of Pittsburgh, Pittsburgh, Pennsylvania, United States of America; 2 Integrative Molecular and Biomedical Sciences Graduate Program, Baylor College of Medicine, Houston, Texas, United States of America; 3 Center for Research on Health Care Data Center, University of Pittsburgh, Pittsburgh, Pennsylvania, United States of America; 4 Department of Pediatrics, Children’s Hospital of Pittsburgh, Pittsburgh, Pennsylvania, United States of America; 5 Institute for Clinical Research Education, University of Pittsburgh, Pittsburgh, Pennsylvania, United States of America; 6 Division of General Internal Medicine, Department of Medicine, University of Pittsburgh, Pittsburgh, Pennsylvania, United States of America; 7 Pittsburgh Heart, Lung, Blood and Vascular Medicine Institute, University of Pittsburgh, Pittsburgh, Pennsylvania, United States of America; Fraunhofer Research Institution of Marine Biotechnology, GERMANY

## Abstract

Irreproducibility of preclinical biomedical research has gained recent attention. It is suggested that requiring authors to complete a checklist at the time of manuscript submission would improve the quality and transparency of scientific reporting, and ultimately enhance reproducibility. Whether a checklist enhances quality and transparency in reporting preclinical animal studies, however, has not been empirically studied. Here we searched two highly cited life science journals, one that requires a checklist at submission (*Nature*) and one that does not (*Cell*), to identify *in vivo* animal studies. After screening 943 articles, a total of 80 articles were identified in 2013 (pre-checklist) and 2015 (post-checklist), and included for the detailed evaluation of reporting methodological and analytical information. We compared the quality of reporting preclinical animal studies between the two journals, accounting for differences between journals and changes over time in reporting. We find that reporting of randomization, blinding, and sample-size estimation significantly improved when comparing *Nature* to *Cell* from 2013 to 2015, likely due to implementation of a checklist. Specifically, improvement in reporting of the three methodological information was at least three times greater when a mandatory checklist was implemented than when it was not. Reporting the sex of animals and the number of independent experiments performed also improved from 2013 to 2015, likely from factors not related to a checklist. Our study demonstrates that completing a checklist at manuscript submission is associated with improved reporting of key methodological information in preclinical animal studies.

## Introduction

Preclinical biomedical research is essential for the development of breakthrough medical therapies. The successful transition from basic research to clinical trials, however, is challenging. Less than 5% of the articles published in the top six life science journals were successfully translated into clinical use despite claims of important clinical implications [1]. Although human and animal biology are expected to be different, this alone does not explain the low rate of successful translation from animal studies to human trials. One pervasive concern is the low level of reproducibility of the results reported in preclinical studies [2, 3], where reproducibility generally refers to “a phenomenon that can be predicted to recur even when experimental conditions may vary to some degree [4].” Although the term has been used differently in biostatistics [5], experimental biological scientists frequently use “reproducibility” to describe the ability to obtain the same scientific findings utilizing similar methods on identical test material by an independent observer [4, 6, 7]. The low level of reproducibility in biomedical experimental research has been attributed, in part, to variations in the quality of reporting of methods and results. Therefore, one proposed solution to this matter is to improve the quality and transparency of reporting of preclinical research findings [8].

In 2014, the National Institutes of Health (NIH) held a joint workshop with leading science publishing groups on the issue of rigor and reproducibility; the consensus was a set of principles emphasizing rigorous statistical analysis, transparency in reporting, and data/material sharing [9]. Specifically, they recommended that journals use a checklist during the editorial processing to ensure transparent reporting of key methodological and analytical information [8, 10]. Many major journals have endorsed these principles and guidelines [11, 12], and since 2013, some have incorporated a checklist in their editorial processing, with different strategies, ranging from an internal checklist during the editorial process to a publicly available reporting checklist authors are required to complete at each manuscript submission.

This was not the first time checklists were employed to improve outcomes. Specifically, the quality of reporting randomized controlled trials (RCTs) also improved with a checklist adopted through the Consolidated Standards of Reporting Trials (CONSORT) statement [13]. Furthermore, a checklist helps to increase general awareness of the problem, leading to better performance [14, 15]. A surgical safety checklist also has been proven to significantly reduce peri-operative morbidity and mortality [16, 17]. We hypothesized that a checklist provided to authors at the time of manuscript submission would improve the quality and transparency in reporting of preclinical studies.

The objectives of this study are to (1) evaluate the current quality of reporting in preclinical biomedical research in highly cited life science journals, and (2) assess the effectiveness of incorporating a pre-submission checklist on the quality and transparency of reporting key methodological and analytical information.

## Materials and methods

### Types of preclinical studies included

All original research articles reporting new data based on animal experiments published in two highly cited life science journals, *Cell* and *Nature*, were included. We limited the scope of our study to articles reporting *in vivo* animal experiments, excluding articles solely based on *in vitro* cell studies.

Both *Cell* and *Nature* possessed the highest impact factors in Journal Citation Reports 2013, and share a similar scope. *Cell* and *Nature* standards and requirements for reporting animal studies are presented in Table 1. Both journals endorse the Principles and Guidelines for Reporting Preclinical Research recommended by NIH [9] and encourage authors and reviewers to refer to reporting standards such as the ARRIVE guidelines [10]. *Cell* guidelines recommend that submitting authors report key methodological and analytical information, while *Nature* guidelines additionally require that a checklist be completed prior to peer review. *Nature* developed their own checklist based on NIH guidelines and implemented it in May 2013. *Nature* also requests a revised checklist to be included with every revision of the manuscript, reflecting any new experiments performed in the interim. A manuscript is sent for review only after the checklist is received. Conversely, *Cell* does not use or require authors to complete a checklist regarding preclinical animal studies, per the policy across the Cell Press journals. The above editorial procedures were confirmed with editors of *Cell* and *Nature*. Therefore, we chose *Nature* as the intervention and *Cell* as the comparator to examine the effect of a checklist on quality and transparency in reporting preclinical animal studies.

**Table 1:** Evaluation of journal standards and requirements as of July 2015.

	*Cell*	*Nature*
Is the Journal a member of endorsing Associations, Journals, and Societies?	Yes	Yes
Does the journal contain a section in the Information for Authors regarding journal's policies for statistical analysis and guidelines for rigorous reporting of study design?	Yes	Yes
**Statistics:** Requirement for statistics to be fully reported in the paper? Statement of statistical test used for each relevant figure presented? Statement of exact value of N for each relevant figure presented? Statements for data definition of center, dispersion and precision measures (e.g., mean, median, SD, SEM, confidence intervals)?	Recommended	Required
	Recommended	Required
	Recommended	Required
	Required	Required
**Replicates:** Investigators are required to report how often each experiment was performed? Investigators are required to report sufficient information about sample collection to distinguish between independent biological data points and technical replicates?	Recommended	Required
	Recommended	Required
**Randomization:** Statements whether the samples were randomized and specify method of randomization, at a minimum for all animal experiments?	Recommended	Required
**Blinding:** Statements whether experimenters were blind to group assignment and outcome assessment, at a minimum for all animal experiments?	Recommended	Required
**Sample size estimation:** Require authors to state whether an appropriate sample size was computed when the study was being designed and include the statistical method of computation. If no power analysis was used, include how the sample size was determined.	Recommended	Required
**Inclusion and exclusion criteria:** Require authors to clearly state the criteria that were used for exclusion of any data or subjects, if any were excluded?	Recommended	Required
**Animals:** Require reporting source, species, strain, sex, age, husbandry, inbred and strain characteristics of transgenic animals? Require ethical governing committee approval of animal use?	Recommended	Required
	Required	Required
**Standards:** Encourage the use of community-based standards (such as nomenclature standards and reporting standards like ARRIVE), where applicable? Provide a checklist form that is made public to authors and reviewers during submission?	Yes	Yes
	No	Yes

### Selection of studies

Studies were selected if they included (1) reports of *in vivo* animal experiments, (2) animal experiments involving mammals, and (3) original research articles. Editorial, commentaries, news, or review articles were excluded. Also original articles reporting only *in vitro* experiments without animal studies were excluded.

### Search methods for identification of studies

**Fig 1:**
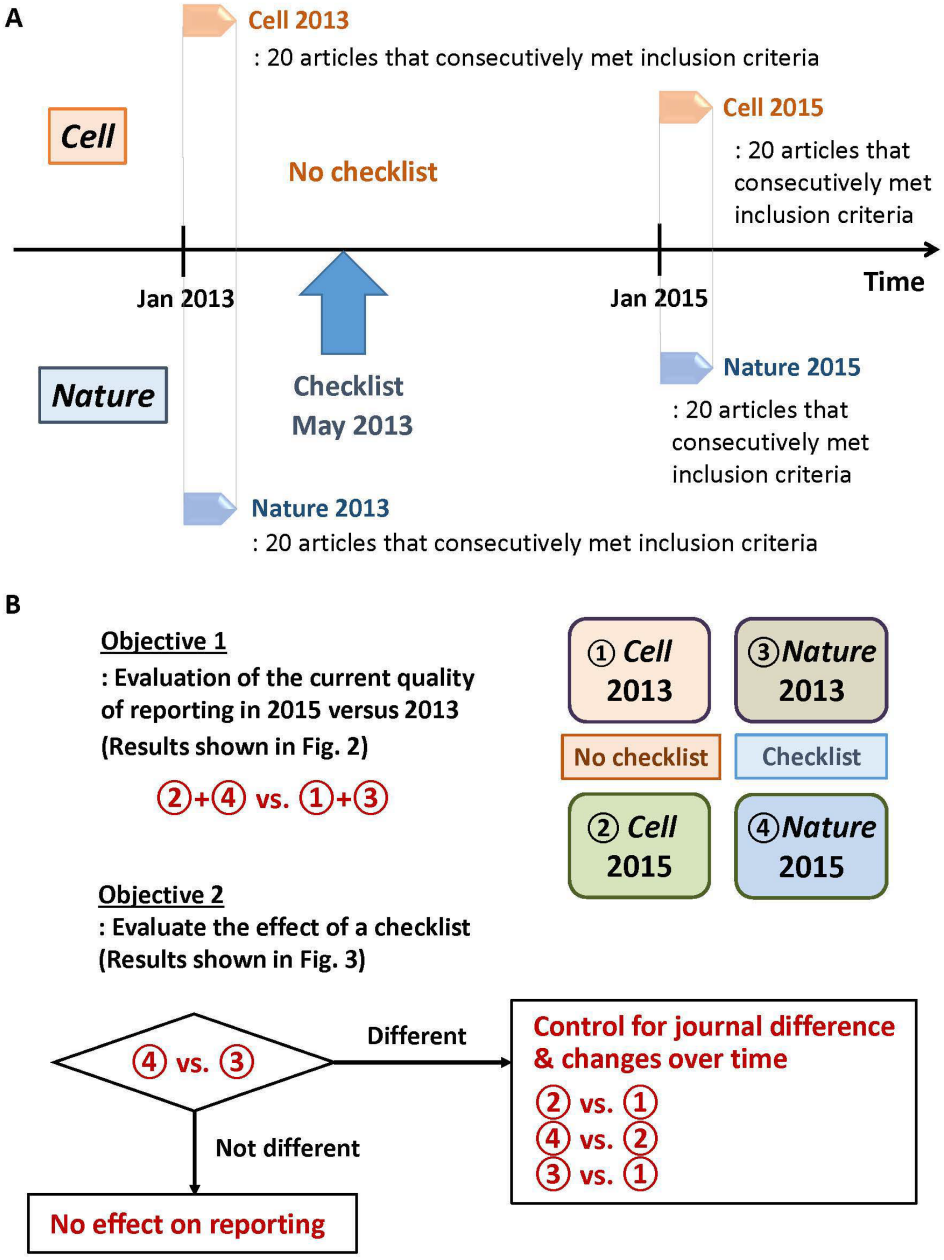
Outline of the study. (A) Selection of articles: Twenty consecutive articles that met the inclusion criteria among those published beginning in January for both 2013 and 2015 in *Nature* (one that implemented a pre-submission checklist) and *Cell* (one that did not) journals. This represents articles from periods of time before and after the implementation of the checklist in May 2013. (B) Flow of the analysis: To examine whether quality of reporting has improved over time, the degree of key information reported in 2015 was compared to that in 2013 in both journals combined (Objective 1). To assess whether a checklist is associated with improved quality in reporting, we first compared the changes over time observed in *Nature* (④ vs. ③). If there was significant difference, we compared time “2015 vs. 2013” in *Cell* (② vs. ①) and *Nature* vs. *Ce*ll within 2013 (③ vs. ①) and 2015 (④ vs. ②) to adjust for differences between journals and changes over time in reporting (Objective 2).

We searched the table of contents from the websites of *Cell* and *Nature*. 943 articles were screened to identify 20 consecutive articles within each journal, meeting the inclusion criteria among those published beginning in January 2013 and January 2015 (Fig 1A, a total article of 80). January 2015 was chosen as the post-checklist period based on the assumption that 1.5 years would provide enough time to implement a new intervention, the checklist, and to keep parallel timeframes. Two authors (TFO or JZ) screened the titles and the abstracts based on the above inclusion criteria. If the title and the abstract were not clear to determine the article’s fulfillment of the inclusion criteria, the full-text of the article was retrieved. If uncertainty remained regarding the selection of an article, another independent reviewer (JSL) made a final decision.

The number of articles, twenty per group, was determined *a priori* based on sample-size estimation (n = 19 per each group). This estimation was based on the assumption that a journal that requires completion of a checklist at the time of submission (i.e., *Nature*) results in increased adherence to transparency reporting guidelines compared to a journal that recommends similar principles and guidelines for reporting preclinical research but does not require the completion of a specific checklist at the time of submission (i.e., *Cell*). On a scale of 2-1-0 for each question (Table 2), we assumed that the difference between the two journals would be 0.5 points per each question. Under the assumption of standard deviation (SD) = 0.7, power = 0.8, and alpha = 0.05, an n = 19 per group was calculated.

**Table 2:** Data abstraction form to assess the quality and transparency in reporting.

	Ratings		
	2	1	0
**Description of Animals**:			
Source	Yes, all reported	Yes, some reported	No, not reported
Species	Yes, all reported	Yes, some reported	No, not reported
Strain	Yes, all reported	Yes, some reported	No, not reported
Sex	Yes, all reported	Yes, some reported	No, not reported
Age	Yes, all reported	Yes, some reported	No, not reported
Inbred and strain characteristics of genetically modified animals detailed?	Yes, all reported	Yes, some reported	No, not reported
	N/A (genetically modified animals not used)		
Identification of committee approving the animal experiments?		Yes	No
**Statistics**:			
Define statistical test used for each relevant figure presented?	Yes, all reported	Yes, some reported	No, not reported
Define test as one-sided or two-sided?	Yes, all reported	Yes, some reported	No, not reported
Exact value of N for each relevant figure presented?	Yes, all reported	Yes, some reported	No, not reported
Definition of center, dispersion and precision measures in figures (e.g., mean, median, SD, SEM, confidence intervals)?	Yes, all reported	Yes, some reported	No, not reported
**Replicates**: Do investigators report how many times experiments were performed independently?	Yes, all reported	Yes, some reported	No, not reported
**Replicates**: Do investigators distinguish biological independent data points and technical replicates?	Yes, all reported	Yes, some reported	No, not reported
**Randomization**: Do authors include a statement whether the samples were randomized even if no randomization was used?	Yes, reported and performed	Yes, reported but not performed	No, not reported
**Blinding**: Do authors include a statement whether experimenters were blinded even if no blinding occurred?	Yes, reported and performed	Yes, reported but not performed	No, not reported
**Sample size estimation**: Do authors include a statement of sample size estimation?	Yes, reported and performed	Yes, reported but not performed	No, not reported

### Data collection

Two authors (SH, TFO, JZ, JPP, or JHY) were randomly assigned to each manuscript to independently evaluate quality and transparency in reporting by utilizing an electronic data abstraction form (Table 2). The items in the form were selected from the consensus of an NIH workshop on the issue of rigor and reproducibility [8, 9]. The form consists of 16 questions to evaluate whether the study meets the core set of reporting standards for rigorous study design. A rating of 2, 1, or 0 was given and corresponded to whether information pertaining to each question was reported for all, some, or none of the data presented, respectively. For the information regarding whether experiments were randomized, blinded, or how the sample size was determined, a rating of 2, 1, 0 corresponded to reported and performed, reported but not performed, and not reported, respectively (Table 2). Reviewers examined the full text of the articles, including figures and tables in addition to supplemental information and references. Even if the information was not present in the full text or supplemental information, an article received credit for reporting information as long as it was available in an article that authors referenced. Any disagreements between the two reviewers were resolved by discussion between the two reviewers. If disagreements were not resolved by discussion, a third independent reviewer made a final decision. After resolving all possible discrepancies by discussion or a third independent reviewer, the final data were downloaded in an electronic file from the database and analyzed.

### Statistical analysis

To examine whether transparency in reporting improved over time, we compared the degree of key information reported in 2015 to that in 2013 in the two journals combined. To assess whether the presence of a checklist had an impact, we compared the changes over time observed in *Nature* with those observed in *Cell*. The flow of the analysis is shown in Fig 1B. Given the sample size and the categorical variables, Fisher exact test was performed for our analyses. We did not correct for multiple comparisons because the hypotheses are independent and sample size was relatively small. All statistical analyses were two-sided, and performed using Stata Statistical Software: Release 14.1 (StataCorp. 2015. College Station, TX: StataCorp LP).

## Results

Two highly cited life science journals, *Nature* (one that requires a checklist at submission) and *Cell* (one that does not), were searched to identify original research articles reporting new data based on *in vivo* animal experiments. A total of 943 articles published from January 1 to February 14 in 2013 and 2015 were examined through a pre-defined search strategy to identify 80 articles that consecutively met our inclusion criteria (see Materials and methods). The number of articles was decided *a priori* based on sample-size estimation. Eighty articles that were reviewed for further analysis are listed in S1 Table. The outline of the study is shown in Fig 1.

### Changes in reporting over time

**Fig 2:**
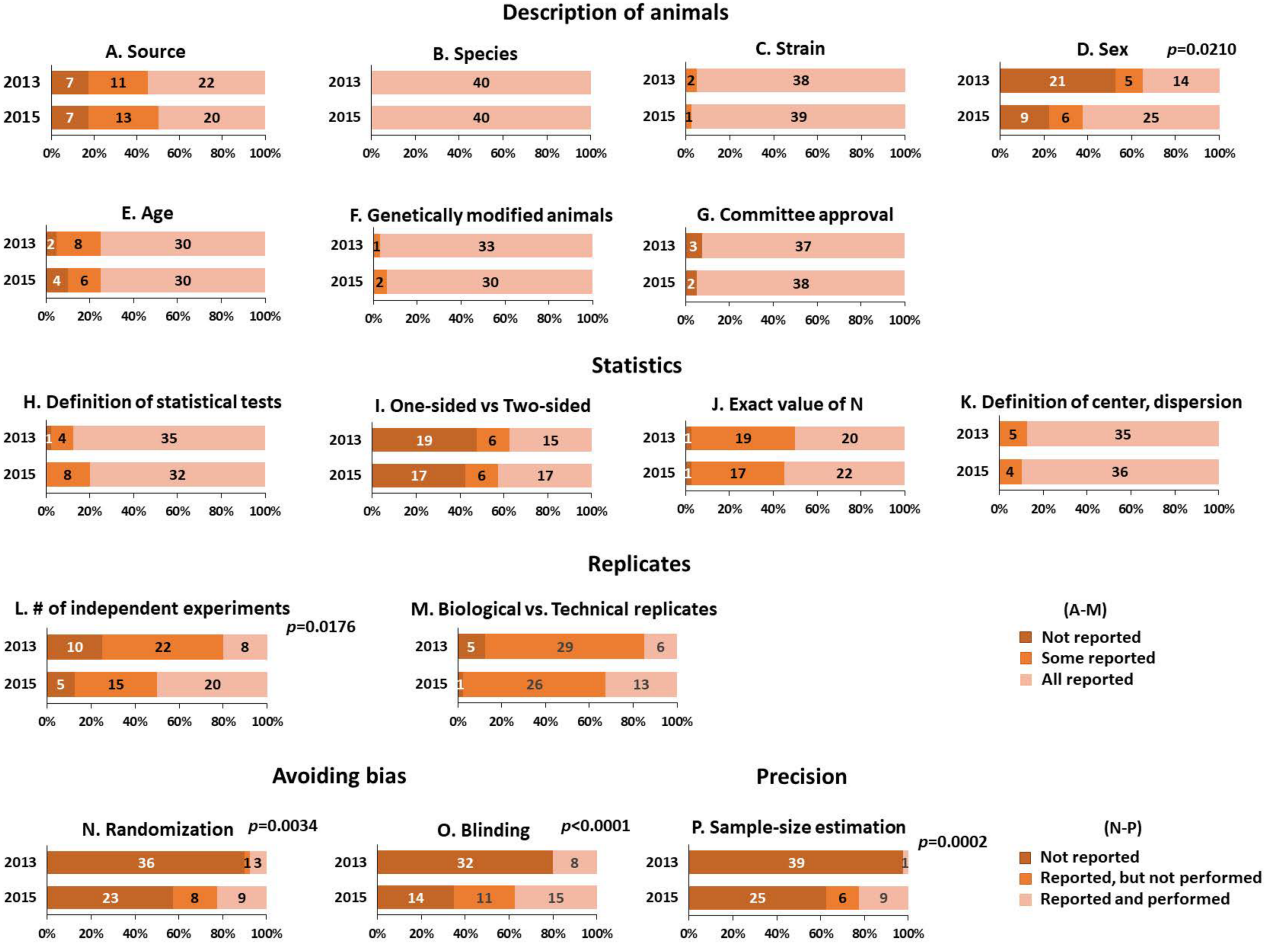
Distribution of reporting study designs across time. The distributions of the reporting status are presented in stacked bar graphs. The numbers inside the stacks are the number of articles corresponding to each percentage. The data for 2013 and 2015 are the total numbers of articles assessed from *Cell* and *Nature* within a given year. Fisher exact test was performed to assess the difference in reporting each methodological across time. Significant *P* values (< 0.05) are provided.

The reporting status of both journals combined between 2013 and 2015 is presented in Fig 2. Overall, the description of study animals was reported (all or some) in more than 80% of the articles in 2013 and 2015, including source, species, strain, age, characterization of genetic modification, and statement of approval by an institutional committee (Fig 2A–2C and 2E–2G). A recent study showed that only 50% of the animal-based studies published in 2014 reported both age and sex variables [18]. Consistent with this, our study showed 42.5% (34 out of 80 articles) reported both sex and age of the study animals completely in 2013 and 2015 (32.5% in 2013, and 52.5% in 2015, respectively). Notably, reporting the sex of animals, however, improved in 2015 when compared to 2013 (the percentage of all or some reported 47.5% in 2013, and 77.5% in 2015 respectively, Fig 2D). In 2013 and 2015, most statistics (statistical methods used, the exact value of sample size, and definition of center and dispersion) were all or some reported in at least 80% of articles examined (Fig 2H, 2J, and 2K). However, whether the statistical tests used were one-sided or two-sided was not reported in approximately 50% (47.5% in 2013, and 42.5% in 2015, respectively, Fig 2I).

**Fig 3:**
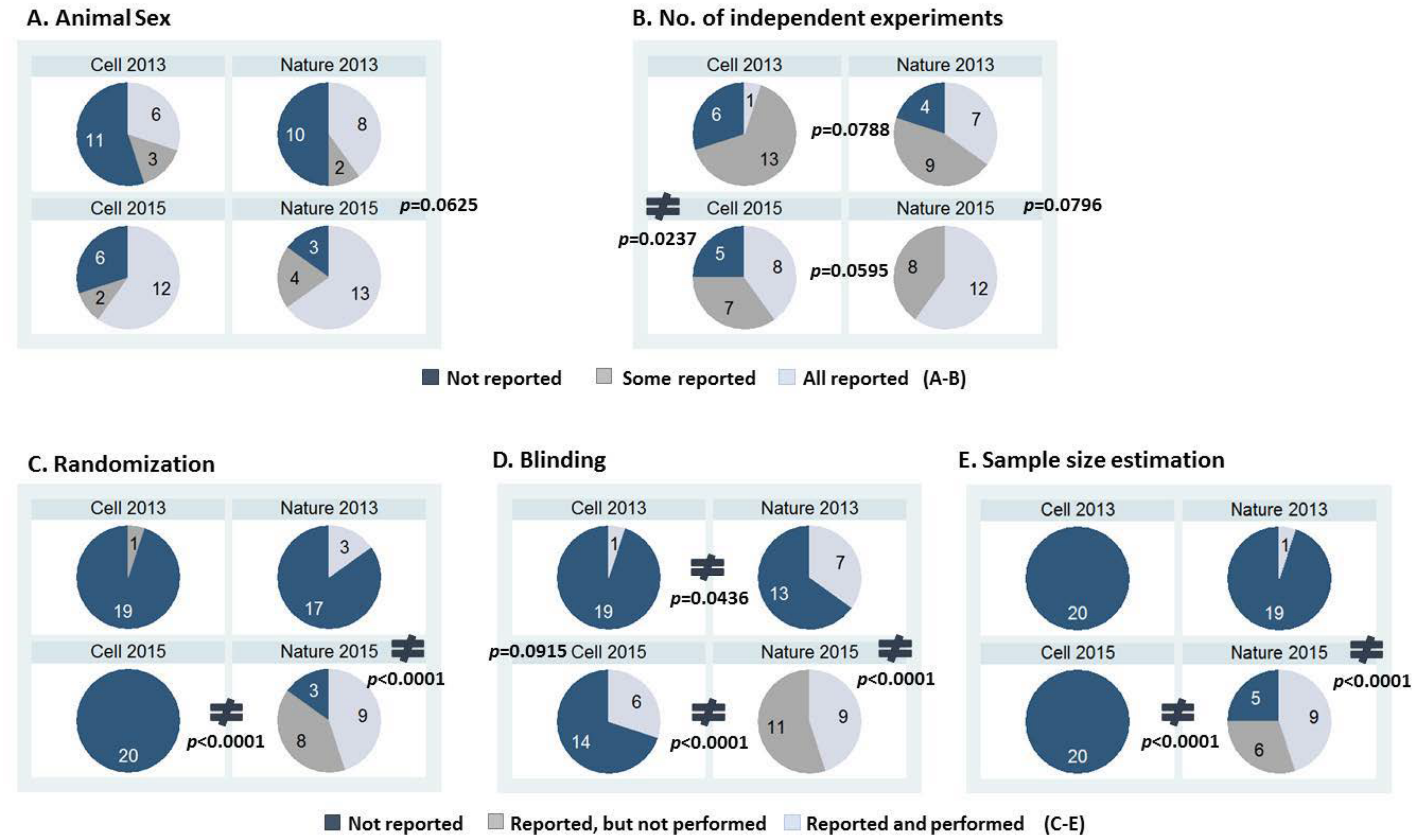
The changes in rigorous reporting of study designs by a checklist. The numbers inside the pie charts are the number of articles corresponding to each category. *P* values < 0.10 using Fisher exact test are provided to compare time 2015 vs. 2013 within the intervention (*Nature*) or the comparison (*Cell*) group, or to compare intervention vs comparison group within 2013 and 2015, respectively. ≠ is shown where comparisons between the touching two groups are significantly different with *P* < 0.05.

Information regarding the number of independent experiments performed, and whether authors distinguished biological versus technical replicates were all or some reported in at least 75% of the articles in 2013 (Fig 2L and 2M). Reporting the number of independent experiments performed improved in 2015 compared to 2013 (*P* = 0.0176). On the other hand, whether experiments were randomized, blinded, or how the sample size was determined were stated in 20% or less of articles in 2013, and improved by 2015 (Fig 2N–2P). Specifically, whether experiments were randomized was reported in 10% of articles in 2013, versus 42.5% in 2015. The information on blinding was reported 20% in 2013, and improved to 65% in 2015. Also, how sample size was determined was stated in 2.5% of articles in 2013, versus 37.5% in 2015. Thus, collective reporting of the following five pieces of methodological information in the two journals significantly improved in 2015 compared to 2013: (1) sex of the animal (Fig 2D); (2) number of independent experiments performed (Fig 2L); (3) randomization (Fig 2N); (4) blinding (Fig 2O); and (5) sample size estimation (Fig 2P).

### Differences between *Cell* and *Nature*

We next evaluated the reporting of methodological information in *Nature* and *Cell* separately to determine whether changes in reporting behavior over time are different by journals. The analyses for the above five methodological information are presented in Fig 3. Other methodological information that is not shown in Fig 3 were not differently reported between *Nature* articles published in 2015 compared to those in 2013 (S2 Table). Reporting the sex of animals did not change over time in each journal (*P* = 0.1626 and *P* = 0.0625 in *Cell* and *Nature*, respectively, Fig 3A), suggesting the improvement in reporting the sex of the animal across time in Fig 2D is unlikely attributable to solely the implementation of a pre-submission checklist.

Reporting the number of independent experiments was statistically different from 2013 to 2015 within *Cell* (*P* = 0.0237) but not within *Nature* (*P* = 0.0796, Fig 3B). However, the reporting of independent experiments in the two journals were not different when compared within 2013 (*P* = 0.0788) and 2015 (*P* = 0.0595) (Fig 3B). This suggests that the improvement in reporting the number of independent experiments performed across time (Fig 2L) is likely attributable to factors not related to a checklist.

On the other hand, the intervention group (*Nature)* showed a significantly greater improvement than the comparison group (*Cell*) in reporting randomization, blinding, and sample-size estimation (Fig 3C–3E). Specifically, the frequency of reporting randomization status was improved from 15% in 2013 to 85% in 2015 within *Nature*, while 5% to 0% within *Cell*. The reporting of whether experiments were blinded, improved from 35% to 100% in *Nature*, but only from 5% to 30% in *Cell*. How sample size was determined was not reported at all in *Cell* both in 2013 and 2015, but the reporting frequency was improved to 75% from 5% in *Nature* after a checklist. Notably, of the articles that were compliant with a checklist, 47% reported that they did not perform randomization, 55% did not perform blinding, and 40% did not perform sample size estimation. These results indicate that incorporation of a checklist did make an impact on the reporting of those study details. Interestingly, reporting the status of blinding in the intervention group (*Nature*) was different from that in the comparison group (*Cell*) in both 2013 (*P* = 0.0436) and 2015 (*P* < 0.0001) (Fig 3D). Despite the difference in reporting of blinding in *Nature* versus *Cell* prior to the checklist, a checklist further improved reporting of blinding because the intervention group (*Nature*) but not the comparison group (*Cell*) improved reporting of this information across time.

We did not correct significance levels for multiple comparisons because we compared the distribution of outcomes in only two groups and did not conduct post hoc tests such as multiple comparison analysis testing in ANOVA (Analysis of variance). We performed a series of Fisher’s exact tests for 16 items simultaneously, but it is not typical to control for the false-positive error rate when performing less than 50 independent tests as doing so over-adjusts [19, 20]. Nonetheless, the level of significance required by the Bonferroni adjustment is (0.05/16) = 0.003125. The improvement in reporting of randomization, blinding and sample-size estimation from 2013 to 2015 within *Nature* remain statistically significant after the adjustment, providing us with the same inferences.

## Discussion

Our study shows that the reporting of important details such as sample size estimation and whether experiments were randomized or blinded improved to a greater degree when a checklist is required, compared with when it is not. Specifically, with a mandatory checklist, the relative frequency of reporting the three methodological information were improved by at least 65%. However, without a checklist it was only improved at most by 25% (absolute difference). In addition, there was overall improvement from 2013 to 2015 in the reporting of sex of animals and number of independent experiments performed in preclinical animal studies. Our results suggest the latter is a time effect, and likely from factors not related to a checklist, such as increased public awareness of transparency in reporting over time.

Reproducibility in biomedical research has been an emerging issue in the scientific community. The sporadic, anecdotal concern [21] was reinforced by alarming reports from pharmaceutical companies [2, 3] that they were able to reproduce results from less than 25% of published preclinical studies. A recent survey showed that 90% of researchers agreed that there is a crisis of reproducibility [22]. Considerable discussion and effort aimed at improving reproducibility continue to engage scientists. However, when the published results cannot be reproduced, it does not always mean that original findings are incorrect. It could simply be that the description of the original experiments was not detailed enough for others to successfully reproduce the findings. To increase transparency and improve reproducibility, the scientific community has endorsed guidelines and provided recommendations for reporting animal research, including the ARRIVE (Animals in Research: Reporting *In Vivo* Experiments) guidelines [8, 10]. Compliance with these guidelines remains low several years later [23]. The less-than-optimal compliance is likely multi-factorial, stemming from reasons such as impracticality of the guidelines for some studies [23] or simply lack of awareness of its existence. Nonetheless, the scientific community needs effective and simple tools to improve reporting of preclinical animal research.

A checklist is an efficient tool to reduce errors and has been successfully implemented in the reporting of RCTs and medical field [13, 14, 17]. For example, the World Health Organization’s Patient Safety Programme developed a surgical safety checklist consisting of 19 items that requires an oral confirmation by the surgical team at the completion of several key steps during the perioperative period [24]. Implementation of the checklist was associated with significantly reduced postoperative complications and mortality [16, 17]. Also, reporting in RCTs improved after adopting the CONSORT checklist [13, 25]. A journal policy endorsing the CONSORT Statement seems to be helpful for improving the reporting of trials published in medical journals [26], especially when journals promote the use of CONSORT checklist by including it in the journal’s “information to authors” section or by requiring authors or manuscript reviewers to complete the checklist [27]. Likewise, some life science journals instituted the use of a checklist during their editorial process to improve transparency in reporting of preclinical biomedical research. However, whether such a checklist improves quality and transparency in reporting of key methodological information in the setting of preclinical research is not known.

Our study shows that a required checklist at the time of manuscript submission improves the reporting of certain methodological information, such as randomization, blinding, and sample-size estimation, in preclinical *in vivo* animal studies. The reporting of this information was poor at baseline in 2013 but improved with incorporation of a checklist. It has been emphasized that randomization, blinding, and sample-size estimation are important for reducing bias in animal studies [28], yet these aspects of study design remain inadequately reported in preclinical research [29]. Specifically, the reporting of those methodological information in cardiovascular preclinical research was poor over the past 10 years [30], which is essentially in accord with our results. This inadequate reporting in preclinical studies contrasts with that in human clinical trials. Many preclinical biomedical studies test their hypotheses at multiple levels with different approaches to try to increase the probability of their findings show an effect, while most human clinical trials do not because of feasibility and ethical limitations. Although methodological tools to reduce bias, such as randomization and blinding, and tools to reduce imprecision such as sample-size estimation have been considered more critical in clinical trials, basic researchers may underestimate the importance of these tools, and instead utilize varied approaches to testing their hypotheses in their preclinical research. A checklist may be a simple and effective tool to remind researchers of the importance of these tools and thus improve the reporting of the information. It is possible that an improvement in the reporting of this information does not translate directly to an improvement in the actual quality of the studies, due to the concern of false/inaccurate reporting (e.g. claiming randomization when there was none). We found, however, that approximately half of the manuscripts that were compliant with the checklist indicated not using these tools, suggesting a substantial amount of truthful reporting of this information.

We also noted that certain methodological information that is generally expected to be completely reported in biomedical research is only partially reported. For example, in approximately half of the articles that we examined in 2013 and 2015, the exact values of sample sizes (N) were not reported for all the data presented. Also, 20% of the articles did not report all the statistical tests that were used. These are areas we have identified that hold opportunities for further improving the reporting of study design and methodology.

Implementing a checklist as a part of the editorial process for journals may improve transparency and the reporting of key information, but this occurs only at a late stage where the experiments have already been conducted and the data submitted. Ideally, a checklist should be used earlier in the research process to avoid experimental errors that would cause a study not to be of publishable quality in the first place. *Nature*’s checklist accomplishes this to a degree, as it was available online for authors to use as a reference when designing experiments. Another concern raised previously is that rigid guidelines may restrict creativity in the early stages of basic research [31]. The main objective of some studies is to describe novel observations of unique phenomena, and certain methodology, such as blinding, randomization, or sample-size estimation, cannot be practically applied for all experiments. At this exploratory rather than confirmatory stage, a checklist may be utilized as a reminder for researchers to simply report whether these methodologies were performed or not.

Our study design may not provide the strongest level of evidence to support our conclusion that a mandatory checklist improves the quality of reporting preclinical animal studies. It is generally considered that the evidence from systematic reviews of RCTs or well-designed RCTs provides the highest level of evidence [32, 33]. Randomization is the best approach to assess causality and minimize confounding by achieving balance between intervention and control groups, however, it is often not feasible to conduct randomization. Quasi-experimental design is an alternative approach to assess causality between an intervention and an outcome without randomization [34, 35]. It requires a concurrent comparison group that is similar to an intervention group with regard to all factors that might affect outcomes except the exposure to the intervention of interest. In our case, it is practically impossible to randomly assign an intervention (checklist) in a journal to address our research question. Therefore, we employed instead a quasi-experimental design that provides the next strongest level of evidence. We identified a concurrent comparison group (*Cell*) that has similar scope and rigorous standards of reporting as *Nature*. Both journals possess the highest impact factors in Journal Citation Reports 2013. We recognize that impact factors may not be the most accurate measure for quality of studies, but at the same time, it is not known precisely what the factors are that affect the quality of reporting methodological and analytical information in preclinical animal studies. Having such a comparison group allowed us to capture the possible outcomes if the checklist had not been implemented. If we only measured the outcomes in *Nature* in 2013 (pre-) and 2015 (post-checklist), measuring and analyzing additional outcomes over time prior to 2013 (time-series analysis) is critical to evaluate the effect of a mandatory checklist. With the before/after approach, any observed improvement in reporting outcomes could be simply the continuation of the improvement over time, independent of a mandatory checklist. On the other hand, a time-series analysis without a concurrent comparison still cannot control for potential confounding factors that are time-varying such as secular trends [34]. As a way to overcome the challenge, we incorporated *Cell* as a concurrent comparison (quasi-experimental design) and thus were able to capture the changes to reporting, over the same period of time, that are not attributable to a mandatory checklist. The frequencies of reporting randomization, blinding, and sample-size estimation were at least three-fold higher when the checklist was used, compared to when it was not. The baseline frequencies of reporting the methodological information in early 2013 before *Nature* implemented a mandatory checklist were not significantly different between the two journals except reporting of blinding (35% in *Nature* versus 5% in *Cell*, p = 0.0436). Even for the reporting of blinding, the degree of improvement in 2015, when compared to 2013, was much greater in *Nature* (65%) than in *Cell* (25%, absolute difference). Hence, we concluded that a checklist can be said to improve the quality of reporting certain methodological information in preclinical studies. While preparing this manuscript, a study protocol on a similar research question with similar study design (quasi-experimental) as ours was published [36]. Although their study will be assisted by the Nature Publishing Group, it will be interesting to compare their results to ours.

## Conclusions

In conclusion, a checklist that is publicly available, and that authors must complete at the time of manuscript submission, improves the quality of reporting preclinical animal studies. Independent of the checklist, the overall quality of reporting data also improved over time since irreproducibility of studies has gained the attention of the scientific community. A checklist may be a simple, practical, and effective tool to promote the quality of scientific reporting.

## Supporting information

S1 Table The list of articles included in this study. (PDF)

S2 Table Quality of reporting across time in each journal: Percent (%). (PDF)
