# Supplementary material for: A checklist is associated with increased quality of reporting preclinical biomedical research: A systematic review
Source: PLoS One. 2017 Sep 13;12(9):e0183591. doi: 10.1371/journal.pone.0183591 (PMC5597130; doi:10.1371/journal.pone.0183591)
Supplement: S1 Table — (PDF) [file pone.0183591.s001.pdf]

Supplementary Materials:

S1 Table. The list of articles included in this study.

Cell 2013	*Cell* 2015	*Nature* 2013	*Nature* 2015
[1-20]	[21-40]	[41-60]	[61-80]

1. Cang C, Zhou Y, Navarro B, Seo YJ, Aranda K, Shi L, et al. mTOR regulates lysosomal ATP-sensitive two-pore Na(+) channels to adapt to metabolic state. Cell. 2013;152(4):778-90. doi: 10.1016/j.cell.2013.01.023. PubMed PMID: 23394946; PubMed Central PMCID: PMCPMC3908667.

2. Chen W, Han C, Xie B, Hu X, Yu Q, Shi L, et al. Induction of Siglec-G by RNA viruses inhibits the innate immune response by promoting RIG-I degradation. Cell. 2013;152(3):467-78. doi: 10.1016/j.cell.2013.01.011. PubMed PMID: 23374343.

3. Gerber A, Esnault C, Aubert G, Treisman R, Pralong F, Schibler U. Blood-borne circadian signal stimulates daily oscillations in actin dynamics and SRF activity. Cell. 2013;152(3):492-503. doi: 10.1016/j.cell.2012.12.027. PubMed PMID: 23374345.

4. Gomez JA, Wapinski OL, Yang YW, Bureau JF, Gopinath S, Monack DM, et al. The NeST long ncRNA controls microbial susceptibility and epigenetic activation of the interferon-gamma locus. Cell. 2013;152(4):743-54. doi: 10.1016/j.cell.2013.01.015. PubMed PMID: 23415224; PubMed Central PMCID: PMCPMC3577098.

5. Kamberov YG, Wang S, Tan J, Gerbault P, Wark A, Tan L, et al. Modeling recent human evolution in mice by expression of a selected EDAR variant. Cell. 2013;152(4):691-702. doi: 10.1016/j.cell.2013.01.016. PubMed PMID: 23415220; PubMed Central PMCID: PMCPMC3575602.

6. Kourrich S, Hayashi T, Chuang JY, Tsai SY, Su TP, Bonci A. Dynamic interaction between sigma-1 receptor and Kv1.2 shapes neuronal and behavioral responses to cocaine. Cell.

2013;152(1-2):236-47. doi: 10.1016/j.cell.2012.12.004. PubMed PMID: 23332758; PubMed Central PMCID: PMCPMC4159768.

7. Ma L, Tao Y, Duran A, Llado V, Galvez A, Barger JF, et al. Control of nutrient stress-induced metabolic reprogramming by PKCzeta in tumorigenesis. Cell. 2013;152(3):599-611. doi: 10.1016/j.cell.2012.12.028. PubMed PMID: 23374352; PubMed Central PMCID: PMCPMC3963830.

8. Masaki T, Qu J, Cholewa-Waclaw J, Burr K, Raaum R, Rambukkana A. Reprogramming adult Schwann cells to stem cell-like cells by leprosy bacilli promotes dissemination of infection. Cell. 2013;152(1-2):51-67. doi: 10.1016/j.cell.2012.12.014. PubMed PMID: 23332746; PubMed Central PMCID: PMCPMC4314110.

9. Mukhopadhyay S, Wen X, Ratti N, Loktev A, Rangell L, Scales SJ, et al. The ciliary G-protein-coupled receptor Gpr161 negatively regulates the Sonic hedgehog pathway via cAMP signaling. Cell. 2013;152(1-2):210-23. doi: 10.1016/j.cell.2012.12.026. PubMed PMID: 23332756.

10. Oh YS, Gao P, Lee KW, Ceglia I, Seo JS, Zhang X, et al. SMARCA3, a chromatin-remodeling factor, is required for p11-dependent antidepressant action. Cell. 2013;152(4):831-43. doi: 10.1016/j.cell.2013.01.014. PubMed PMID: 23415230; PubMed Central PMCID: PMCPMC3633087.

11. Ostuni R, Piccolo V, Barozzi I, Polletti S, Termanini A, Bonifacio S, et al. Latent enhancers activated by stimulation in differentiated cells. Cell. 2013;152(1-2):157-71. doi: 10.1016/j.cell.2012.12.018. PubMed PMID: 23332752.

12. Schwitalla S, Fingerle AA, Cammareri P, Nebelsiek T, Goktuna SI, Ziegler PK, et al. Intestinal tumorigenesis initiated by dedifferentiation and acquisition of stem-cell-like properties. Cell. 2013;152(1-2):25-38. doi: 10.1016/j.cell.2012.12.012. PubMed PMID: 23273993.

13. Sohn JW, Harris LE, Berglund ED, Liu T, Vong L, Lowell BB, et al. Melanocortin 4 receptors reciprocally regulate sympathetic and parasympathetic preganglionic neurons. Cell. 2013;152(3):612-9. doi: 10.1016/j.cell.2012.12.022. PubMed PMID: 23374353; PubMed Central PMCID: PMCPMC3711728.

14. Solovei I, Wang AS, Thanisch K, Schmidt CS, Krebs S, Zwerger M, et al. LBR and lamin A/C sequentially tether peripheral heterochromatin and inversely regulate differentiation. Cell. 2013;152(3):584-98. doi: 10.1016/j.cell.2013.01.009. PubMed PMID: 23374351.

15. Xu B, Hsu PK, Stark KL, Karayiorgou M, Gogos JA. Derepression of a neuronal inhibitor due to miRNA dysregulation in a schizophrenia-related microdeletion. Cell. 2013;152(1-2):262-75. doi: 10.1016/j.cell.2012.11.052. PubMed PMID: 23332760; PubMed Central PMCID: PMCPMC3556818.

16. Xue Y, Ouyang K, Huang J, Zhou Y, Ouyang H, Li H, et al. Direct conversion of fibroblasts to neurons by reprogramming PTB-regulated microRNA circuits. Cell. 2013;152(1-2):82-96. doi: 10.1016/j.cell.2012.11.045. PubMed PMID: 23313552; PubMed Central PMCID: PMCPMC3552026.

17. Yan J, Xiang J, Lin Y, Ma J, Zhang J, Zhang H, et al. Inactivation of BAD by IKK inhibits TNFalpha-induced apoptosis independently of NF-kappaB activation. Cell. 2013;152(1-2):304-15. doi: 10.1016/j.cell.2012.12.021. PubMed PMID: 23332762; PubMed Central PMCID: PMCPMC3586589.

18. Yildirim E, Kirby JE, Brown DE, Mercier FE, Sadreyev RI, Scadden DT, et al. Xist RNA is a potent suppressor of hematologic cancer in mice. Cell. 2013;152(4):727-42. doi: 10.1016/j.cell.2013.01.034. PubMed PMID: 23415223; PubMed Central PMCID: PMCPMC3875356.

19. Yu Y, Chen Y, Kim B, Wang H, Zhao C, He X, et al. Olig2 targets chromatin remodelers to enhancers to initiate oligodendrocyte differentiation. Cell. 2013;152(1-2):248-61. doi: 10.1016/j.cell.2012.12.006. PubMed PMID: 23332759; PubMed Central PMCID: PMCPMC3553550.

20. Zala D, Hinckelmann MV, Yu H, Lyra da Cunha MM, Liot G, Cordelieres FP, et al. Vesicular glycolysis provides on-board energy for fast axonal transport. Cell. 2013;152(3):479-91. doi: 10.1016/j.cell.2012.12.029. PubMed PMID: 23374344.

21. Anderson DM, Anderson KM, Chang CL, Makarewich CA, Nelson BR, McAnally JR, et al. A micropeptide encoded by a putative long noncoding RNA regulates muscle performance. Cell. 2015;160(4):595-606. doi: 10.1016/j.cell.2015.01.009. PubMed PMID: 25640239; PubMed Central PMCID: PMCPMC4356254.

22. Antal CE, Hudson AM, Kang E, Zanca C, Wirth C, Stephenson NL, et al. Cancer-associated protein kinase C mutations reveal kinase's role as tumor suppressor. Cell. 2015;160(3):489-502. doi: 10.1016/j.cell.2015.01.001. PubMed PMID: 25619690; PubMed Central PMCID: PMCPMC4313737.

23. Bonavita E, Gentile S, Rubino M, Maina V, Papait R, Kunderfranco P, et al. PTX3 is an extrinsic oncosuppressor regulating complement-dependent inflammation in cancer. Cell. 2015;160(4):700-14. doi: 10.1016/j.cell.2015.01.004. PubMed PMID: 25679762.

24. Bourane S, Grossmann KS, Britz O, Dalet A, Del Barrio MG, Stam FJ, et al. Identification of a spinal circuit for light touch and fine motor control. Cell. 2015;160(3):503-15. doi: 10.1016/j.cell.2015.01.011. PubMed PMID: 25635458; PubMed Central PMCID: PMCPMC4431637.

25. Chan CK, Seo EY, Chen JY, Lo D, McArdle A, Sinha R, et al. Identification and specification of the mouse skeletal stem cell. Cell. 2015;160(1-2):285-98. doi: 10.1016/j.cell.2014.12.002. PubMed PMID: 25594184; PubMed Central PMCID: PMCPMC4297645.

26. Dittmann M, Hoffmann HH, Scull MA, Gilmore RH, Bell KL, Ciancanelli M, et al. A serpin shapes the extracellular environment to prevent influenza A virus maturation. Cell. 2015;160(4):631-43. doi: 10.1016/j.cell.2015.01.040. PubMed PMID: 25679759; PubMed Central PMCID: PMCPMC4328142.

27. Dodd GT, Decherf S, Loh K, Simonds SE, Wiede F, Balland E, et al. Leptin and insulin act on POMC neurons to promote the browning of white fat. Cell. 2015;160(1-2):88-104. doi: 10.1016/j.cell.2014.12.022. PubMed PMID: 25594176; PubMed Central PMCID: PMCPMC4453004.

28. Hine C, Harputlugil E, Zhang Y, Ruckenstuhl C, Lee BC, Brace L, et al. Endogenous hydrogen sulfide production is essential for dietary restriction benefits. Cell. 2015;160(1-2):132-44. doi: 10.1016/j.cell.2014.11.048. PubMed PMID: 25542313; PubMed Central PMCID: PMCPMC4297538.

29. Hofmann JW, Zhao X, De Cecco M, Peterson AL, Pagliaroli L, Manivannan J, et al. Reduced expression of MYC increases longevity and enhances healthspan. Cell.

2015;160(3):477-88. doi: 10.1016/j.cell.2014.12.016. PubMed PMID: 25619689; PubMed Central PMCID: PMCPMC4624921.

30. Huch M, Gehart H, van Boxtel R, Hamer K, Blokzijl F, Verstegen MM, et al. Long-term culture of genome-stable bipotent stem cells from adult human liver. Cell. 2015;160(1-2):299-312. doi: 10.1016/j.cell.2014.11.050. PubMed PMID: 25533785; PubMed Central PMCID: PMCPMC4313365.

31. Jennings JH, Ung RL, Resendez SL, Stamatakis AM, Taylor JG, Huang J, et al. Visualizing hypothalamic network dynamics for appetitive and consummatory behaviors. Cell. 2015;160(3):516-27. doi: 10.1016/j.cell.2014.12.026. PubMed PMID: 25635459; PubMed Central PMCID: PMCPMC4312416.

32. Lee MW, Odegaard JI, Mukundan L, Qiu Y, Molofsky AB, Nussbaum JC, et al. Activated type 2 innate lymphoid cells regulate beige fat biogenesis. Cell. 2015;160(1-2):74-87. doi: 10.1016/j.cell.2014.12.011. PubMed PMID: 25543153; PubMed Central PMCID: PMCPMC4297518.

33. Liu L, Zhang K, Sandoval H, Yamamoto S, Jaiswal M, Sanz E, et al. Glial lipid droplets and ROS induced by mitochondrial defects promote neurodegeneration. Cell. 2015;160(1-2):177-90. doi: 10.1016/j.cell.2014.12.019. PubMed PMID: 25594180; PubMed Central PMCID: PMCPMC4377295.

34. Loo JM, Scherl A, Nguyen A, Man FY, Weinberg E, Zeng Z, et al. Extracellular metabolic energetics can promote cancer progression. Cell. 2015;160(3):393-406. doi: 10.1016/j.cell.2014.12.018. PubMed PMID: 25601461; PubMed Central PMCID: PMCPMC4312495.

35. McDermott DH, Gao JL, Liu Q, Siwicki M, Martens C, Jacobs P, et al. Chromothriptic cure of WHIM syndrome. Cell. 2015;160(4):686-99. doi: 10.1016/j.cell.2015.01.014. PubMed PMID: 25662009; PubMed Central PMCID: PMCPMC4329071.

36. McDonald ME, Li C, Bian H, Smith BD, Layne MD, Farmer SR. Myocardin-related transcription factor A regulates conversion of progenitors to beige adipocytes. Cell. 2015;160(1-2):105-18. doi: 10.1016/j.cell.2014.12.005. PubMed PMID: 25579684; PubMed Central PMCID: PMCPMC4384505.

37. Nieh EH, Matthews GA, Allsop SA, Presbrey KN, Leppla CA, Wichmann R, et al. Decoding neural circuits that control compulsive sucrose seeking. Cell. 2015;160(3):528-41. doi: 10.1016/j.cell.2015.01.003. PubMed PMID: 25635460; PubMed Central PMCID: PMCPMC4312417.

38. Worthley DL, Churchill M, Compton JT, Tailor Y, Rao M, Si Y, et al. Gremlin 1 identifies a skeletal stem cell with bone, cartilage, and reticular stromal potential. Cell. 2015;160(1-2):269-84. doi: 10.1016/j.cell.2014.11.042. PubMed PMID: 25594183; PubMed Central PMCID: PMCPMC4436082.

39. Yan Y, Jiang W, Liu L, Wang X, Ding C, Tian Z, et al. Dopamine controls systemic inflammation through inhibition of NLRP3 inflammasome. Cell. 2015;160(1-2):62-73. doi: 10.1016/j.cell.2014.11.047. PubMed PMID: 25594175.

40. Yang J, Wu Z, Renier N, Simon DJ, Uryu K, Park DS, et al. Pathological axonal death through a MAPK cascade that triggers a local energy deficit. Cell. 2015;160(1-2):161-76. doi: 10.1016/j.cell.2014.11.053. PubMed PMID: 25594179; PubMed Central PMCID: PMCPMC4306654.

41. Alonzo F, 3rd, Kozhaya L, Rawlings SA, Reyes-Robles T, DuMont AL, Myszka DG, et al. CCR5 is a receptor for Staphylococcus aureus leukotoxin ED. Nature. 2013;493(7430):51-5. doi: 10.1038/nature11724. PubMed PMID: 23235831; PubMed Central PMCID: PMCPMC3536884.

42. Arnon TI, Horton RM, Grigorova IL, Cyster JG. Visualization of splenic marginal zone B-cell shuttling and follicular B-cell egress. Nature. 2013;493(7434):684-8. doi: 10.1038/nature11738. PubMed PMID: 23263181; PubMed Central PMCID: PMCPMC3561487.

43. Barry ER, Morikawa T, Butler BL, Shrestha K, de la Rosa R, Yan KS, et al. Restriction of intestinal stem cell expansion and the regenerative response by YAP. Nature. 2013;493(7430):106-10. doi: 10.1038/nature11693. PubMed PMID: 23178811; PubMed Central PMCID: PMCPMC3536889.

44. Chaudhury D, Walsh JJ, Friedman AK, Juarez B, Ku SM, Koo JW, et al. Rapid regulation of depression-related behaviours by control of midbrain dopamine neurons. Nature. 2013;493(7433):532-6. doi: 10.1038/nature11713. PubMed PMID: 23235832; PubMed Central PMCID: PMCPMC3554860.

45. Diehl GE, Longman RS, Zhang JX, Breart B, Galan C, Cuesta A, et al. Microbiota restricts trafficking of bacteria to mesenteric lymph nodes by CX(3)CR1(hi) cells. Nature. 2013;494(7435):116-20. doi: 10.1038/nature11809. PubMed PMID: 23334413; PubMed Central PMCID: PMCPMC3711636.

46. Efeyan A, Zoncu R, Chang S, Gumper I, Snitkin H, Wolfson RL, et al. Regulation of mTORC1 by the Rag GTPases is necessary for neonatal autophagy and survival. Nature. 2013;493(7434):679-83. doi: 10.1038/nature11745. PubMed PMID: 23263183; PubMed Central PMCID: PMCPMC4000705.

47. Gkogkas CG, Khoutorsky A, Ran I, Rampakakis E, Nevarko T, Weatherill DB, et al. Autism-related deficits via dysregulated eIF4E-dependent translational control. Nature. 2013;493(7432):371-7. doi: 10.1038/nature11628. PubMed PMID: 23172145; PubMed Central PMCID: PMCPMC4133997.

48. Haider B, Hausser M, Carandini M. Inhibition dominates sensory responses in the awake cortex. Nature. 2013;493(7430):97-100. doi: 10.1038/nature11665. PubMed PMID: 23172139; PubMed Central PMCID: PMCPMC3537822.

49. Heneka MT, Kummer MP, Stutz A, Delekate A, Schwartz S, Vieira-Saecker A, et al. NLRP3 is activated in Alzheimer's disease and contributes to pathology in APP/PS1 mice. Nature. 2013;493(7434):674-8. doi: 10.1038/nature11729. PubMed PMID: 23254930; PubMed Central PMCID: PMCPMC3812809.

50. Juncadella IJ, Kadl A, Sharma AK, Shim YM, Hochreiter-Hufford A, Borish L, et al. Apoptotic cell clearance by bronchial epithelial cells critically influences airway inflammation. Nature. 2013;493(7433):547-51. doi: 10.1038/nature11714. PubMed PMID: 23235830; PubMed Central PMCID: PMCPMC3662023.

51. Knobloch M, Braun SM, Zurkirchen L, von Schoultz C, Zamboni N, Arauzo-Bravo MJ, et al. Metabolic control of adult neural stem cell activity by Fasn-dependent lipogenesis. Nature. 2013;493(7431):226-30. doi: 10.1038/nature11689. PubMed PMID: 23201681; PubMed Central PMCID: PMCPMC3587167.

52. Kornfeld JW, Baitzel C, Konner AC, Nicholls HT, Vogt MC, Herrmanns K, et al. Obesity-induced overexpression of miR-802 impairs glucose metabolism through silencing of Hnf1b. Nature. 2013;494(7435):111-5. doi: 10.1038/nature11793. PubMed PMID: 23389544.

53. Lee AM, Kanter BR, Wang D, Lim JP, Zou ME, Qiu C, et al. Prkcz null mice show normal learning and memory. Nature. 2013;493(7432):416-9. doi: 10.1038/nature11803. PubMed PMID: 23283171; PubMed Central PMCID: PMCPMC3548047.

54. Qin J, Wu SP, Creighton CJ, Dai F, Xie X, Cheng CM, et al. COUP-TFII inhibits TGF-beta-induced growth barrier to promote prostate tumorigenesis. Nature. 2013;493(7431):236-40. doi: 10.1038/nature11674. PubMed PMID: 23201680; PubMed Central PMCID: PMCPMC4022346.

55. Santini E, Huynh TN, MacAskill AF, Carter AG, Pierre P, Ruggero D, et al. Exaggerated translation causes synaptic and behavioural aberrations associated with autism. Nature. 2013;493(7432):411-5. doi: 10.1038/nature11782. PubMed PMID: 23263185; PubMed Central PMCID: PMCPMC3548017.

56. Senyo SE, Steinhauser ML, Pizzimenti CL, Yang VK, Cai L, Wang M, et al. Mammalian heart renewal by pre-existing cardiomyocytes. Nature. 2013;493(7432):433-6. doi: 10.1038/nature11682. PubMed PMID: 23222518; PubMed Central PMCID: PMCPMC3548046.

57. Tye KM, Mirzabekov JJ, Warden MR, Ferenczi EA, Tsai HC, Finkelstein J, et al. Dopamine neurons modulate neural encoding and expression of depression-related behaviour. Nature. 2013;493(7433):537-41. doi: 10.1038/nature11740. PubMed PMID: 23235822; PubMed Central PMCID: PMCPMC4160519.

58. Volk LJ, Bachman JL, Johnson R, Yu Y, Huganir RL. PKM-zeta is not required for hippocampal synaptic plasticity, learning and memory. Nature. 2013;493(7432):420-3. doi: 10.1038/nature11802. PubMed PMID: 23283174; PubMed Central PMCID: PMCPMC3830948.

59. Vrontou S, Wong AM, Rau KK, Koerber HR, Anderson DJ. Genetic identification of C fibres that detect massage-like stroking of hairy skin in vivo. Nature. 2013;493(7434):669-73.

doi: 10.1038/nature11810. PubMed PMID: 23364746; PubMed Central PMCID: PMCPMC3563425.

60. Weber JN, Peterson BK, Hoekstra HE. Discrete genetic modules are responsible for complex burrow evolution in Peromyscus mice. Nature. 2013;493(7432):402-5. doi: 10.1038/nature11816. PubMed PMID: 23325221.

61. Banks AS, McAllister FE, Camporez JP, Zushin PJ, Jurczak MJ, Laznik-Bogoslavski D, et al. An ERK/Cdk5 axis controls the diabetogenic actions of PPARgamma. Nature. 2015;517(7534):391-5. doi: 10.1038/nature13887. PubMed PMID: 25409143; PubMed Central PMCID: PMCPMC4297557.

62. Barretto RP, Gillis-Smith S, Chandrashekar J, Yarmolinsky DA, Schnitzer MJ, Ryba NJ, et al. The neural representation of taste quality at the periphery. Nature. 2015;517(7534):373-6. doi: 10.1038/nature13873. PubMed PMID: 25383521; PubMed Central PMCID: PMCPMC4297533.

63. Buffie CG, Bucci V, Stein RR, McKenney PT, Ling L, Gobourne A, et al. Precision microbiome reconstitution restores bile acid mediated resistance to Clostridium difficile. Nature. 2015;517(7533):205-8. doi: 10.1038/nature13828. PubMed PMID: 25337874; PubMed Central PMCID: PMC4354891.

64. Cheng CJ, Bahal R, Babar IA, Pincus Z, Barrera F, Liu C, et al. MicroRNA silencing for cancer therapy targeted to the tumour microenvironment. Nature. 2015;518(7537):107-10. doi: 10.1038/nature13905. PubMed PMID: 25409146; PubMed Central PMCID: PMCPMC4367962.

65. Cuskin F, Lowe EC, Temple MJ, Zhu Y, Cameron EA, Pudlo NA, et al. Human gut Bacteroidetes can utilize yeast mannan through a selfish mechanism. Nature. 2015;517(7533):165-9. doi: 10.1038/nature13995. PubMed PMID: 25567280.

66. Deng K, Pertea M, Rongvaux A, Wang L, Durand CM, Ghiaur G, et al. Broad CTL response is required to clear latent HIV-1 due to dominance of escape mutations. Nature. 2015;517(7534):381-5. doi: 10.1038/nature14053. PubMed PMID: 25561180; PubMed Central PMCID: PMCPMC4406054.

67. Faller WJ, Jackson TJ, Knight JR, Ridgway RA, Jamieson T, Karim SA, et al. mTORC1-mediated translational elongation limits intestinal tumour initiation and growth. Nature. 2015;517(7535):497-500. doi: 10.1038/nature13896. PubMed PMID: 25383520; PubMed Central PMCID: PMCPMC4304784.

68. Krupic J, Bauza M, Burton S, Barry C, O'Keefe J. Grid cell symmetry is shaped by environmental geometry. Nature. 2015;518(7538):232-5. doi: 10.1038/nature14153. PubMed PMID: 25673417; PubMed Central PMCID: PMCPMC4576734.

69. Kurtova AV, Xiao J, Mo Q, Pazhanisamy S, Krasnow R, Lerner SP, et al. Blocking PGE2-induced tumour repopulation abrogates bladder cancer chemoresistance. Nature. 2015;517(7533):209-13. doi: 10.1038/nature14034. PubMed PMID: 25470039; PubMed Central PMCID: PMCPMC4465385.

70. Ling LL, Schneider T, Peoples AJ, Spoering AL, Engels I, Conlon BP, et al. A new antibiotic kills pathogens without detectable resistance. Nature. 2015;517(7535):455-9. doi: 10.1038/nature14098. PubMed PMID: 25561178.

71. Liu D, Xu H, Shih C, Wan Z, Ma X, Ma W, et al. T-B-cell entanglement and ICOSL-driven feed-forward regulation of germinal centre reaction. Nature. 2015;517(7533):214-8. doi: 10.1038/nature13803. PubMed PMID: 25317561.

72. Peretti D, Bastide A, Radford H, Verity N, Molloy C, Martin MG, et al. RBM3 mediates structural plasticity and protective effects of cooling in neurodegeneration. Nature.

2015;518(7538):236-9. doi: 10.1038/nature14142. PubMed PMID: 25607368; PubMed Central PMCID: PMCPMC4338605.

73. Reniere ML, Whiteley AT, Hamilton KL, John SM, Lauer P, Brennan RG, et al. Glutathione activates virulence gene expression of an intracellular pathogen. Nature. 2015;517(7533):170-3. doi: 10.1038/nature14029. PubMed PMID: 25567281; PubMed Central PMCID: PMC4305340.

74. Stensola T, Stensola H, Moser MB, Moser EI. Shearing-induced asymmetry in entorhinal grid cells. Nature. 2015;518(7538):207-12. doi: 10.1038/nature14151. PubMed PMID: 25673414.

75. Strait RT, Posgai MT, Mahler A, Barasa N, Jacob CO, Kohl J, et al. IgG1 protects against renal disease in a mouse model of cryoglobulinaemia. Nature. 2015;517(7535):501-4. doi: 10.1038/nature13868. PubMed PMID: 25363774; PubMed Central PMCID: PMCPMC4342786.

76. Vaughan AE, Brumwell AN, Xi Y, Gotts JE, Brownfield DG, Treutlein B, et al. Lineage-negative progenitors mobilize to regenerate lung epithelium after major injury. Nature. 2015;517(7536):621-5. doi: 10.1038/nature14112. PubMed PMID: 25533958; PubMed Central PMCID: PMCPMC4312207.

77. Venkatanarayan A, Raulji P, Norton W, Chakravarti D, Coarfa C, Su X, et al. IAPP-driven metabolic reprogramming induces regression of p53-deficient tumours in vivo. Nature. 2015;517(7536):626-30. doi: 10.1038/nature13910. PubMed PMID: 25409149; PubMed Central PMCID: PMCPMC4312210.

78. Wang D, Cai C, Dong X, Yu QC, Zhang XO, Yang L, et al. Identification of multipotent mammary stem cells by protein C receptor expression. Nature. 2015;517(7532):81-4. doi: 10.1038/nature13851. PubMed PMID: 25327250.

79. Xue S, Tian S, Fujii K, Kladwang W, Das R, Barna M. RNA regulons in Hox 5' UTRs confer ribosome specificity to gene regulation. Nature. 2015;517(7532):33-8. doi: 10.1038/nature14010. PubMed PMID: 25409156; PubMed Central PMCID: PMC4353651.

80. Zuo W, Zhang T, Wu DZ, Guan SP, Liew AA, Yamamoto Y, et al. p63(+)Krt5(+) distal airway stem cells are essential for lung regeneration. Nature. 2015;517(7536):616-20. doi: 10.1038/nature13903. PubMed PMID: 25383540.
