# Supplementary material for: A checklist is associated with increased quality of reporting preclinical biomedical research: A systematic review
Source: PLoS One. 2017 Sep 13;12(9):e0183591. doi: 10.1371/journal.pone.0183591 (PMC5597130; doi:10.1371/journal.pone.0183591)
Supplement: S2 Table — (PDF) [file pone.0183591.s002.pdf]

S2 Table. Quality of reporting across time in each journal: percent (%)

		Cell			*Nature*		
		**2013**	**2015**	***p*-value** [a]	**2013**	**2015**	***p*-value** [b]
**Description of animals**							
**Source**	Not reported	15	15	0.9011	20	20	> 0.9999
	Some reported	20	30		35	35	
	All reported	65	55		45	45	
**Species**	All reported	100	100	N/A	100	100	N/A
**Strain**	Some reported	0	0	N/A	10	5	> 0.9999
	All reported	100	100		90	95	
**Sex**	Not reported	55	30	0.1626	50	15	0.0625
	Some reported	15	10		10	20	
	All reported	30	60		40	65	
**Age**	Not reported	5	15	0.4128	5	5	0.1818
	Some reported	15	25		25	5	
	All reported	80	60		70	90	
**Genetic modification** [c]	Some reported	0	11	0.4866	6	0	> 0.9999
	All reported	100	89		94	100	
**Committee approval**	Not reported	10	5	> 0.9999	5	5	> 0.9999
	Reported	90	95		95	95	
**Statistics**							
**Definition of statistical tests**	Not reported	5	0	0.2351	0	0	> 0.9999
	Some reported	10	30		10	10	
	All reported	85	70		90	90	
**One-sided vs. Two-sided**	Not reported	60	55	> 0.9999	35	30	> 0.9999
	Some reported	20	20		10	10	
	All reported	20	25		55	60	
**Exact value of sample size**	Not reported	5	5	> 0.9999	0	0	> 0.9999
	Some reported	50	45		45	40	
	All reported	45	50		55	60	
**Definition of center/dispersion**	Some reported	20	0	0.1060	5	20	0.3416
	All reported	80	100		95	80	
**Replicates**							
**# of independent experiments**	Not reported	30	25	0.0237	20	0	0.0796
	Some reported	65	35		45	40	
	All reported	5	40		35	60	
**Biological vs. Technical replicates**	Not reported	15	5	0.2044	10	0	0.3341
	Some reported	80	70		65	60	
	All reported	5	25		25	40	
**Avoiding Bias**							
**Randomization**	Not reported	95	100	> 0.9999	85	15	<.0001
	Reported, but not performed	5	0		0	40	
	Reported and performed	0	0		15	45	

**Blinding**	Not reported	95	70	0.0915	65	0	<.0001
	Reported, but not performed	0	0		0	55	
	Reported and performed	5	30		35	45	
**Precision**							
**Sample-size estimation**	Not reported	100	100	N/A	95	25	<.0001
	Reported, but not performed	0	0		0	30	
	Reported and performed	0	0		5	45	

n=20 for each group (Total n=80); *P* values by Fisher's exact test; N/A, not applicable
[a] Comparison between *Cell* 2013 and *Cell* 2015; [b] Comparison between *Nature* 2013 and *Nature* 2015; [c] n=16 for *Cell* 2013, n=18 for *Cell* 2015, n=18 for *Nature* 2013, and n=14 for *Nature* 2015
